# Integrating Palliative Care by Virtue of Diplomacy; A Cross-sectional Group Interview Study of the Roles and Attitudes of Palliative Care Professionals to Further Integrate Palliative Care in Europe

**DOI:** 10.34172/ijhpm.2020.211

**Published:** 2020-11-23

**Authors:** Jelle van Gurp, Jeroen van Wijngaarden, Sheila Payne, Lukas Radbruch, Karen van Beek, Ágnes Csikós, Marlieke Herder-van der Eerden, Jeroen Hasselaa, InSup-C (FP7) Research Consortium

**Affiliations:** ^1^Department of IQ healthcare, Radboud University Nijmegen Medical Centre, Nijmegen, The Netherlands.; ^2^Erasmus School of Health Policy & Management, Health Service Management and Organisation, Erasmus University Rotterdam, Rotterdam, The Netherlands.; ^3^International Observatory on End of Life Care, Division of Health Research, Lancaster University, Lancaster, UK.; ^4^Department of Palliative Medicine, University Hospital Bonn, Bonn, Germany.; ^5^Department of Radiation-Oncology and Palliative Medicine, University Hospital Gasthuisberg, Leuven, Belgium.; ^6^Faculty of Medicine, Institute of Family Medicine, University of Pécs Medical School, Pécs, Hungary.; ^7^Department of Anesthesiology, Pain and Palliative Medicine, Radboud University Nijmegen Medical Centre, Nijmegen, The Netherlands.

**Keywords:** Integration of Care, Palliative Care, Virtue Ethics, Diplomacy, Integrated Care

## Abstract

**Background: **Palliative care involves the care for patients with severe and advanced diseases with a focus on quality of life and symptom management. Integration of palliative care with curative and/or chronic care is expected to lead to better results in terms of quality of life and reduced costs. Although initiatives in different countries in Europe choose different structures to integrate care, they face similar challenges when it comes to creating trust and aligning visions, cultures and professional values. This paper sets out to answer the following research question: what roles and attitudes do palliative care professionals need to adopt to further integrate palliative care in Europe?

**Methods: **As part of the European Union (EU)-funded research project InSup-C (Integrated Supportive and Palliative Care). (2012-2016), 19 semi-structured group interviews with 136 (palliative) care professionals in 5 European countries (Germany, the United Kingdom, Belgium, the Netherlands, Hungary) were conducted. A thematic analysis was conducted.

**Results: **Integration of palliative care calls for diplomatic professionals that can bring a cultural shift: to get palliative care, with its particular focus on the four dimensions (physical, psychological, social, spiritual), integrated into historically established medical procedures and guidelines. This requires (a) to find an entrance (for telling a normative story), and (b) to maintain and deepen relationships (in order to build trust). It means using the appropriate words and sending a univocal team message to patients and being grateful, modest, and aiming for a quiet revolution with curation oriented healthcare professionals.

**Conclusion:** Diplomacy appears to be essential to palliative care providers for realizing trust and what can be defined as normative integration between palliative and curative and/or chronic medicine. It requires a practical wisdom about the culture and goals of regular care, as well as keeping a middle road between assimilating with values in regular medicine and standing up for the basic values central to palliative care.

## Background

**Key Messages**

** Implications for policy makers**

- This paper illustrates the relevance of aligning missions and work values (normative integration) of palliative and curative medicine.
- More particularly it focuses on the virtue of diplomacy as a prerequisite for palliative care representatives to move palliative care towards the centre of medicine.
- This paper shows the opportunities and pitfalls when taking up diplomacy as a key virtue for integration.
- Policy-makers could further integration by ensuring that palliative care teaching is widely available to new and junior healthcare professionals, so that they are instilled with a broader perspective on, and a different language about, care for patients with advanced and incurable diseases.

**Implications for the public**

With better integration of palliative care with curative and/or chronic care, the general public will benefit from broader-oriented healthcare professionals who are better equipped to combine their medical specialism with a more holistic and person-oriented care approach. This is expected to result in more patient-centred care, especially during serious illness and in the last phase of life. Moreover, further integration will contribute to better collaboration between medical specialists, more continuity of care for patients, and fluid transitions between curative and palliative care. Patient-physician conversations and shared decision-making processes will become less ambiguous as the involved healthcare professionals will have their values and norms better aligned. Improved normative integration also means that professionals will experience less insecurity, implying that they can focus more on patient care and patients’ quality of life.

Palliative care is an approach that aims to improve the quality of life of patients and their families facing the problems associated with life-threatening illness.^1^ Integration of palliative care with curative and/or chronic care has a positive impact on, among others, symptom control, quality of life, continuity of care, costs and caregiver burden.^2,3^ Based on a literature review, followed by a consensus meeting with European palliative care experts,^4^ integrated palliative care can be defined as bringing together administrative, organisational, clinical and services aspects of treatment and care in order to achieve continuity of care between all actors involved in the care network of patients in need of palliative care. The literature shows that continuity of care is about the treatment of patients by the right care giver, at the right moment, at the right place (patient logistics), about the availability of information at the right time (information transfer) about continuity in the content of the treatment between involved professionals (content of care) and about the timely availability of resources and materials.^5,6^ Integrated palliative care aims to achieve quality of life and a well-supported dying process for the patient and the family in collaboration with all the caregivers, paid and unpaid.^7^ Despite increasing evidence of the positive effects of early involvement of palliative care in serious illness trajectories, healthcare practices still often remain fragmented.^8^ For example, continuity of care between healthcare professionals and general practitioner (GP) involvement in the last week is considered crucial for patients to be able to die at the preferred place.^9^ However, a substantial proportion of patients (19%-29%) with cancer die in hospitals in the Netherlands and Belgium, usually the non-preferred place of death.^10^ Transfers of patients in the last phase of life put a high burden on patients and their families, but also involve high costs for society. As the number of people suffering and dying from cancer and other, usually chronic diseases (for example chronic heart failure, chronic obstructive pulmonary disease, and diabetes) in Europe is expected to increase in the coming years,^11^ the lack of timely palliative care remains an important focus point for public health policy-makers. While there is broad agreement that at least two levels of palliative care are necessary to provide good coverage and quality of palliative care (general approach and specialist level), there is still discussion on how these are effectively implemented in the current healthcare structures.^12-14^

 Due to different historical and cultural contexts, integration of palliative care initiatives and national healthcare systems throughout Europe is characterized by a variety of challenges. On the administrative level, for example, palliative care in Europe struggles with being appropriately funded, but in very different ways.^15^ Although European countries walked different paths to try to bring together administrative, organisational, clinical and services aspects of palliative care, they also face similar challenges. While they may choose different structures and processes to integrate care, they all need to align interests, values and social relations between caregivers.^16^ In an earlier paper we already showed that integration of palliative care especially depends on the recognition of palliative care values (by other care givers and patients), on trust (between palliative care specialists and other care givers), and on the recognition of palliative care having added value (by other caregivers and patients).^17^ Whereas existing healthcare structures are, by and large, organized to find the causes of (sometimes chronic) diseases as well as treatments aiming for cure or at least prolongation of life, palliative care is a more care-oriented, holistic patient approach that should help the patient to live as comfortable as possible during serious, life-threatening illness and towards the end of his/her life, ideally reaching inner peace and acceptance of mortality.^18^ Palliative caregivers that strive for integration are described as performing balancing act between protecting the original palliative care values and opening up to other medical disciplines without compromising these early ideals in an unnecessary medicalization of death and dying.^14^ The research question of this study is therefore: what roles, attitudes, and tasks do healthcare professionals working in palliative care need to adopt to further integrated palliative care in Europe? To answer these questions group interview data with palliative care professionals from 19 integrated palliative care initiatives in five European countries were studied as part of the European Union (EU) funded research program InSup-C (Integrated Supportive and Palliative Care). Our data collection focuses on integration at the micro-level (primary care process) and meso level (organizational and professional), not at the macro level integration of the healthcare system.

## Methods

 Group interviews were considered the appropriate method for gaining insight in the various perspectives of participants on their roles in the building and formalisation of European integrated palliative care initiatives. Led by native researchers, group interviews offer an opportunity for multi-perspective discussions with a group of knowledgeable healthcare professionals affiliated with a particular initiative.^19^ Compared to individual interviews, group interviews produce more varied, elaborated (personal views, critical feedback, intersubjective perspectives, contextual data) data that is representative for the integrated palliative care initiative. An ‘everyday’ form of group communication with open questions as stimuli is thought to “tell [a lot] of what people know or experience.”^20^

###  Recruitment

 For this study, part of the EU-funded research project InSup-C on integrated palliative care,^7^ a total of twenty-three integrated palliative care initiatives were selected from five European countries (a convenient sample): Germany, the United Kingdom, Belgium, the Netherlands, Hungary. Nevertheless the differences in national palliative care practices and healthcare systems in these countries, this study looked for common experiences by healthcare professionals working in or closely with integrated palliative care initiatives. The selected initiatives were characterized by a focus on (*a*) local palliative care collaboration between at least two different organizations and (*b*) provision of direct patient care by a multidisciplinary group of professionals.^7^ Four initiatives withdrew from the group interview study due to lack of time or opportunity to further cooperate. In the period from May 2015 to January 2016, 19 group interviews were conducted: 2 in Belgium, 4 in the Netherlands, 4 in the United Kingdom, 4 in Germany, and 5 in Hungary Detailed characteristics of the participating initiatives are described in another paper (Table).^21^

**Table:** Schematic Overview of Themes

**Main Theme: The Virtue of Diplomacy**	
A palliative care diplomat has at least two tasks in order to move palliative care from the periphery to the center of medicine. Either to coexist but preferably to enter into a synergetic relationship	
1. To find an entrance (for telling a normative story about palliative care, ie, a coherent story on how palliative care should be performed)	2. To maintain and deepen relationships (in order to build trust with patients and other caregivers)
Being a diplomat around patients is characterized by: doing practical care work while being open to a conversation on palliative care knowing when to talk (and when not to talk) to patients about bad prognoses while, at the same time, introducing a palliative care perspective using the appropriate words giving room for patients to express their requests, but also being honest about unrealistic requests taking responsibility for making sure the team sends a univocal message	
Diplomacy with healthcare professionals is characterized by: Investing in personal connections To be of service Requiring approval for being involved Being grateful for referrals Being modest in the presence of others – aiming for the quiet revolution Being continuously available/approachable Fully sharing of information with other disciplines	

 Contact persons within the initiatives as well as patients participating in an individual interview study^22^ were asked to designate healthcare professionals that were connected to the initiative. Those healthcare professionals were then invited to join the group interviews. For each initiative 15–25 team members were invited, as the participation rate on group interviews is usually quite low. In this way, 6 to 10 attendees per group were reasonably guaranteed. As a result of this recruitment strategy, the group interviews contained both representatives from palliative and curative care (please see Supplementary file 1; details of participants). However, the professionals working in curative care were usually already familiar with the integrated care initiative and, in general, quite positive about palliative care. They could mostly be considered boundary workers, causing these group interviews to be skewed towards critically appraising the process of integration in palliative care from the perspective of those already more or less involved in palliative care.

###  Data Collection

 An interview protocol with initial open questions as well as suggestions for probing structured the interviews. This protocol was developed by JvW and JvG drawing on propositions extracted from the World Health Organization (WHO) definition of palliative care^23^ and theoretical concepts connected to integrated care (ie, content of care, patient flow, information logistics, availability of resources and material).^4,24-26^ After a first feedback round with the international research group, the preliminary interview protocol was improved and then tested for its feasibility in the United Kingdom and Germany. In a first international meeting, the initial experiences with the protocol were discussed after which the international research group finalized the interview protocol (Supplementary file 2). This meeting was imperative to guarantee a uniform group interview procedure, irrespective of language and/or culture. All moderators had sufficient experience with group interviews. The moderators were accompanied by a second researcher from the national research team in order to guarantee completeness and sufficient depth (JH, SH, AC, KVB, LLD).

 Preceding the group interviews, the participants provided verbal consent. The group interviews were mostly conducted on the site of the integrated palliative care initiatives and in the national languages, lasted on average 90 minutes (range 60-120 minutes), and were audio-recorded and later transcribed verbatim. Data were collected between May 2015 and January 2016.

###  Data Analysis

 To facilitate a uniform analysis of the interview transcripts, it was decided that the Dutch research team would analyze the group interview data. Therefore, the transcripts that were not available in Dutch or English were translated into English by professional translators (Hungarian and German interviews). To compensate for the loss of contextual knowledge as a consequence of the translation and the analysis performed by a monocultural research group, each country provided extensive additional memos with characteristics of the initiatives as well as of the particular national healthcare systems.

 Researchers JvW, JvG, and MHvdE performed a stepwise inductive qualitative analysis in line with a thematic analysis,^27^ resulting in a cohesive overview of themes, subthemes, and patterns. The analysts were particularly sensitive to the topic of study due to their professional experience and knowledge of the respective literature^28^: research and theory about integrated care (JvW), healthcare ethics and anthropology (JvG), international public health and anthropology (MHvdE), palliative care, ethics and health policy (JH). Without a theoretical framework, but with sensitive researchers, the analysis started with open coding of the raw data. In a first step, JvW and JvG open coded six transcripts from four countries. After an exploratory discussion about the codes, the researchers then independently applied “comparative analysis” between similarly coded fragments to logically cluster codes, then labelled these clusters with higher-order concepts (themes), and searched for relationships between these concepts. In this way, an iterative coding scheme consisting of central themes, subthemes, and exemplary quotes was realized that was discussed until consensus about the coding scheme was reached. In a second step, researcher MHvdE was trained in working with the scheme. Then JvW, JvG, and MHvdE (re)coded all transcripts using CAQDAS ATLAS.ti. Newly emerging themes were first discussed and then added to the coding scheme.

 In a third, parallel step the validity of the codes and coding scheme was tested by means of multiple peer discussions: the fit and relevance of the coding scheme was a central topic in two plenary discussions during international project meetings. In addition, the Dutch team (JH, MHvdE) organized one-on-one (face-to-face or Skype) discussions with each country to understand themes/topics in relation to the national healthcare context.

###  Quality Quarantees

 The validity of the concepts was guaranteed through using “cross-referenced multiple opinions”^29^ in the group interview data, triangulation between national cases, and international data triangulation. The peer discussions not only contributed to the validity of concepts, but also guaranteed that the national characteristics were considered with regard to particular health system organizations. The international discussions made sure that cultural blind spots were revealed. The coding scheme was considered to be of high quality when it appeared relevant for all international cases and was able to interpret and explain the care processes in various initiatives.

## Results

 The analysis of the group interviews shows that palliative care professionals in all five countries are continuously applying the virtue of diplomacy in their work with both patients and other healthcare professionals. The interviewees explained that professionals working in palliative care are aiming for a cultural shift: to get palliative care, with its particular focus on the four dimensions (physical, psychological, social, spiritual), integrated into historically established medical procedures and guidelines for seriously and/or chronically ill people, preferably as early in the disease trajectory as possible. Based on the data, this diplomacy work can be unfolded into two particular tasks, namely to find an entrance (in order to be able to tell a normative story about palliative care) and to maintain and deepen relationships (in order to build trust with patients and caregivers) (Table).

 Respondents reported that professionals working in palliative medicine are passionate professionals who believe in relating to other specialties and patients in such a way that they can share their ideals and vision of palliative care. In relating to others, language and labelling is essential. For instance, palliative care professionals continue to reflect on whether to use the label palliative care or labels such as supportive care and comfort care, where the latter seem to be less confronting and definitive for patients and other professionals. Diplomacy, seen as continuous tactful management of, often unequal, relationships between different institutes, helps to maintain a fine balance between being a supportive palliative care service that suggests a holistic outlook on patient treatment and care, and the deeply felt motivation to convince patients and healthcare professionals that the last phase of life can be lived to the fullest in all its aspects.

###  Being a Diplomat Around Patients

 The interviewed healthcare professionals reported that seriously ill patients are not necessarily convinced of the value of palliative care. Instead, they have to show this value in working with patients, especially for those who “stubbornly stick to [frequently considered curative] treatments” (B2,3). An open and holistic, but also practical attitude is the usual means by which professionals find an entrance with patients. Instead of explaining and rationalizing the general concept of palliative care, healthcare professionals build a practical base for further palliative care collaboration with a patient through focusing first on particular and urgent needs of patients. This practical collaboration provides the basis for building a shared normative frame of reference. They live it rather than conceptualize it.

 “The patients needs are in the forefront. […] on the so-called first visit, [the team members] introduce themselves, they might even tell the patient what we represent [but not necessarily]” [G3].

####  Diplomacy Requires Appropriate Timing and Use of Words

 To find an entrance with patients and keep their trust, the tactful timing of communicating difficult truths together with introducing a palliative care perspective is key. The initial visit – as early in the disease process as possible – is an important first step. This timing is considered one of the most important and difficult tasks of palliative care providers.

 “*Of all the things we do day in day out, I consider this our most difficult and biggest task. What information at what time” *[G3].

*“… you notice when people want to discuss [end-of-life]. Often I say: ‘your wife is also scared. How are things going to be in the nearby future?’ And then: WHAM, you get the whole story. […] They know, then, that I can be trusted. That there can be openness, honesty” *[NL3].

 The interviewed healthcare professionals explain that picking the right moment to bring further bad news concerning prognosis and introducing a palliative care perspective is much easier within oncology than when supporting people with organ failure, because the course of cancer and future scenarios are easier to predict.

*“I say that in case of COPD [chronic obstructive pulmonary disease] and chronic heart failure they cannot see how serious it is, and that they practically have the life expectancy of cancer patients or even worse, in some cases. This is absolutely not in the public mind, so when I communicate this, it is very difficult to accept for them, even for relatives as well”* [HU1].

 When it comes to introducing a palliative care perspective, the choice of words is essential. For the broad public, words such as ‘palliative’ and ‘hospice’ often relate to death knocking on the door. Professionals are still looking for alternative terms.

*“I wonder if the term ‘palliative care’ could be threatening for certain patients. Can’t we look for terminology that is less stirring […] ‘palliative’ equals ‘dying’ for people”* [B1].

 The interviewees also mentioned an important downside to an open attitude combined with speaking in veiled terms. Diplomacy should not result in becoming too flexible with patients: providing too much room for unrealistic requests and leaving decisions too much with the patient and family.

*“… taking the flexibility too far […] and saying, ‘Well, so you let us know when … there’s a problem.’ […] patients and carers don’t know how bad a problem needs to get to justify calling someone”* [UK5].

####  Diplomacy Comes With Responsibility

 Interviewees report that in their integrated care initiatives there is only limited formalization concerning who is going to take responsibility for telling, with caution, difficult truths to a patient. Interviewees reported that often uncertainty exists about who is responsible for such communication, potentially disregarding the patient who is lost in the middle. Occasionally, this results in healthcare professionals who bear only limited responsibility breaking bad news to patients at badly-timed moments. In addition, interviewees experience that in multidisciplinary care settings patients check with different professionals whether the ‘truth’ that has been communicated to them personally, is a shared truth among these professionals. If they find out it is not, professionals risk patients feeling highly insecure and losing their carefully built up relationships.

*“And had that consultation … if it had been shared with another health professional involved in the care, maybe they could have picked that up, because obviously within a chronic disease there’s a limited time … […] And that needs to be passed on to somebody to run with to take further, you know, there’s almost like the fallout from it that needs picking up…” *[UK1].

 Diplomacy With Healthcare Professionals

 In order to find an entrance into the already existing professional networks to spread and implement palliative care ideals, interviewees talked about the need to have the right attitude (diplomacy) for investing in interprofessional connections. Although regulations and accreditation processes increasingly require integration with palliative care, it remains difficult for palliative medicine to get admitted in the traditionally curative oriented, prominent medical specialties (eg, cardiology, oncology). Although it is slowly changing, within these specialties palliative medicine is still often seen as having lower status, is non-scientific and not high-tech, and non-profitable. In building relationships with other professionals, formalization of palliative care in existing healthcare practices is mainly absent.

*“There are no established basics in this area [communication with GPs], and therefore, we always need a great deal of empathy and consideration because we can cause immense damage by failing to communicate*” [G3].

 Some particular specialties and medical cultures (different for the various initiatives) prove to be almost impenetrable, but generally an attitude of being of service helps to find an entrance with other medical specialties. Interviewees talk of getting approval from responsible physicians to be involved with patients, to express gratitude when patients are referred to them, and to be modest in the presence of other medical professionals.

*“I was invited to come and join the multidisciplinary team meetings [of the department of pulmonary diseases], but I wasn’t allowed to say much [in the first year]”* [NL3].

 Palliative care services usually try to be available and approachable as much as possible, but some interviewees mention that being available has its limits. Initiatives in some countries might not have the resources to run a full time (24/7) service due to lack of reimbursement for, for example, out of hours services.

*“(Palliative care) nurses and doctors work on a part-time job basis, so they do not do it full-time. On-call fee is not paid, so you cannot expect them to stay ready to make a third shift and work even at night” *[HU2].

####  Diplomacy With Professionals

 Palliative care professionals are expected to invest in personal connections with other specialists. Such personal connections, the “getting to know each other,” lead to greater trust in each other’s work and assessments and acknowledging each other’s “hang-ups” (UK3). Then, these trustful relationships function as a vehicle for information transfer, the sharing of ideals, and actual collaboration. “Personal liaisons” (NL2), not necessarily following existing pathways or guidelines, guarantee more integrated care, especially in acute and stressful situations.

*“I think it’s going to have to be, you know, building bridges and building that face to face and recognising other people’s…” *[UK3].

*“The only way they got him home was by communication [between all partners involved; hospital and community] – absolutely everybody just came together and this man died where he wanted to be, which was at home in his own bed” *[UK5].

 There needs to be regular communication about the patient’s condition, in a timely manner so that meaningful interaction is possible. Furthermore, information needs to be fully shared. As appeared from the group interview data, these norms were often frustrated: professionals and institutes do not necessarily comply. The data are inconclusive when it comes to explaining why these norms are hard to follow. Interviewees think medical specialists simply disregard the palliative care perspective, older specialists have their routines and forget or are not interested to include other disciplines (HU3 “many of the physicians say, whether they are a surgeon, an internist, or a GP, that because they have had so many dying patients they could deal with them.”), or circumstances (eg, legislation on privacy and data sharing) make it hard for professionals to share their information.

 However, some improvements have been noticed partly because younger specialists tend to be more susceptible to change as a consequence of palliative care education – and because (digital) technologies increasingly support information sharing.

*“I see that more often, that the specialist writes down in the electronic file to call the GP. It strikes me: we’ve fought this [the lack of communication between specialist and GP] for many years, and now it seems to improve”* [NL3].

 It appears from the interviews that palliative care professionals aim for a quiet revolution. As soon as these professionals receive approval to work or be present in other departments, they will try to get the palliative care message across through words (mainly providing feedback, training, and up to date guidelines) and work (when they participate in patient care).

*“We provide constant feedback to the prescribing doctor. When we are on-site with a patient, we provide feedback to the prescriber about possible changes which could assist in optimising the therapy”* [G3]

 Interestingly, the implementation of palliative medicine and/or care remains dependent on the trust factor. Notwithstanding palliative care paragraphs in guidelines and pathways, actual collaboration and integration between medical specialists and palliative care specialists can only come about when mutual trust in interprofessional relationships is established. And it seems to be the palliative care specialist who most of the time has to take the first step.

*“What really advances things is when we know each other personally. It is far more reassuring when we know each other. Not just on an institutional level. And I think this is true in every area. We have an idea of the other’s activities. It is easier to raise certain things, because we have a basic trust” *[G3].

 A potential risk lurks as a continuous adapting and being of service can result in a vulnerable working position. When working on that cultural shift, palliative care specialists aim to be as approachable and easy-going as possible. But such an open and cooperative attitude has to be limited as other specialists might take advantage by ‘dumping’ difficult – but not necessarily palliative care – cases. Palliative care specialists need to remain in charge when it comes to appropriate referral and discharge in order to take care of the right patients at the right time, and also to control the workload and remain flexible.

*“Since, of course we need to be careful when it comes to, say, admittance to the ward, to make certain that our system does not get abused. [...] And then, for example we get a patient who is primarily suffering from depression. At home of course they do not receive good care, but they have a melanoma, which on the other hand is completely stable at the moment. And then they call us to find out if we could undertake their care. [...] That is, we always have to fight even with ourselves to remain true to our mission. So we mustn’t try to do our best only because we are so easy to approach”* [G4].

 Finally, an important experienced down-side of having to rely on personal relationships of trust is that it is quite labor intensive to maintain these relationships. As a consequence palliative care networks only stretch to a limited number of professionals, focusing on specific diseases.

 A lack of univocal and consequent communication concerning the abovementioned topics among healthcare professionals usually causes faulty normative integration between patients and healthcare professionals.

## Discussion

 The group interviews that were at the basis of this article showed that palliative care professionals working on integration with other medical disciplines share a common desire: to promote a holistic outlook on patient treatment and care, and a deeply felt motivation to convince patients and other healthcare professionals that the last phase of life/a life with a serious, life-threatening illness can be lived to the fullest in all its aspects. In order to accomplish this goal palliative care professionals require the virtue of diplomacy. Diplomacy is needed in contacts with both patients and representatives from other medical specialties and ideally results in tactful communication and trust. Following the results, diplomacy should be understood as a virtue that can be learned by going from copying to gaining a “more unified and explanatory understanding of one’s own diplomacy.”^30^ Diplomacy can be learned by looking at teachers and role models who are a little bit more diplomatic, but learners can eventually surpass their teachers when developing their own understanding of their practices.^30^ At least, the diplomatic palliative care expert knows how to react in various healthcare contexts to patients, families and physicians and has tailor-made services and care to offer. As is common with virtues, diplomacy appears together and is intertwined with other virtues (eg, the cardinal virtues courage, practical wisdom, and temperance, and virtues such as modesty and assertiveness).

 The existing healthcare practices are characterized by a culture with curative norms and values, and by people who aspire to reach these goals through virtuous behaviour.^31^ When it comes to integration however, the present culture can be put to the test by – in this case – palliative care and be challenged to change and adapt. In the literature this is referred to as normative integration: “the extent to which mission, work values etc become shared within a system.”^24^ Successful normative integration then results in a new clear mission and vision, which are acknowledged by all and supported by leadership.^24,31^ In this respect, palliative care professionals should be prepared to sometimes assimilate, sometimes confront and sometimes adapt, in order to add their goal to the intrinsic goals of already well-established medical practices. In this process, there is a risk that the particular palliative care perspective is diluted till it becomes no longer recognizable. Diplomacy, as described in our study, can be regarded as a way for palliative care professionals to withstand this risk but it requires having a practical wisdom about the culture and goals of existing practices, as well as keeping the middle road between an obligingness to integrate and fully be of service on the one hand and to stand firm for the basic values within palliative care at the other. With diplomacy will come trust, and trust is the basis for “mutual agreement on purpose and ends, together with a working consensus on the means and practices guiding behaviours.”^31^ In order to break through the hegemony of what is described in the palliative care literature as a “technology driven medicine based on life prolonging interventions,”^32^ we conclude, based on the results, that palliative care specialists chose to work:

- Incognito. They do not discuss their palliative care perspective over and over again with patients, withhold using terms that explicitly refer to palliative care, and try to show their value through practical acts. While getting to know the patient better, palliative care specialists get a feel for when to raise some topics from a palliative care perspective.
- In the service towards other medical specialties. Generally, palliative caregivers are modest and grateful for an entrance. By being too approachable and too open, however, the interviewees emphasized that it is difficult to integrate while at the same time staying true to their original cause and ideals (see also Clark^14^). Palliative care professionals that gained so much trust that they can be steadfast when it comes to appropriate referral, care, and discharge might characterize a successful integration of the intrinsic values and goals of palliative care into an organization’s mission.
- With accurate interprofessional communication. As communication requires reciprocity, it is still dependent on the good will of other specialties (but being diplomatic helps). Modern communication technologies appear to be of use as they, at first sight, help to simplify interprofessional communication. There are, nowadays, more opportunities to share information about patients but practical issues, privacy ethics, and legislation often make this difficult to realize.

This study’s integrated palliative care initiatives seem to aim for a quiet revolution within healthcare organizations. But the actions of those who actually try to integrate palliative care with general medicine as described in this empirical study are still rather covert: careful choice of words is essential in keeping relationships with patients and families, having to be dependent on other specialities to approach a patient, the need for trustful relationships in order to get your ideals across.^33-35^

 Optimal integrated palliative care is still a work in progress. The interviewees, however, emerged as modest, patient, and highly motivated teachers. To further integrated palliative care this kind of teaching has to be widely available to new and junior healthcare professionals, so that the palliative care perspective is quietly added to a curative perspective (the generalist approach).^13^ Palliative care professionals have to accept that they often speaks a specific language and should guard themselves against being too adaptive to the curative goals and language often dominant in existing healthcare practices, whilst being sensitive for mutual gains to support quality of life of patients with advanced and severe diseases. To interviewees it seems important to preserve palliative care’s particular language, although not always pushing it to the foreground. Thus, scientific evidence on palliative care and palliative care protocols seem relevant, especially when gradually incorporated into unique educational material through which young professionals are instilled with a broader perspective on and a different language about care for those who suffer a serious, life-threatening illness. That also means that in current healthcare systems palliative care professionals have to develop a teaching role while remaining diplomats fostering palliative care being part of regular healthcare and healthcare education.

 To conclude, palliative care often takes place in a situation where death and dying are present, although what constitutes a good death can have varying meanings and connotations. Patients faced with their mortality can also show different coping strategies, on a continuum of acceptance to complete denial. Caregivers, therefore, need careful and sensitive communication skills to recognize and to address this, but in practice often implicit language is used (the unspeakable death) that in itself may cause a barrier for an appropriate referral to palliative care.^36^ When the virtue of diplomacy is well-developed, caregivers can subtly work around this unspeakable death and open up possibilities for adequate care for the last phase of life. Relational continuity and entrusted relationships with patients, and also between professional caregivers – who may or may not be open for a palliative care approach with a focus on quality of life rather than an acute care approach – are key caregiver.^22^ This often takes place in a context where family members are highly emotionally involved in the care for their beloved one and that palliative caregivers need to address the needs of the patient as well as the needs of the family in a proactive manner.^37^

###  Limitations

 Healthcare culture is a central concept in this paper, and within a European context healthcare cultures and structures differ vastly. The data for this study came from different European countries, but the results section in this paper might suggest that there’s one dominant European palliative care perspective that needs to be integrated with one dominant (curative) healthcare culture. The argument to present the need for palliative care specialists to be diplomatic in such a specific way is that the data as well as the peer reviews made clear that healthcare professionals in all countries struggle with normative integration with respect to palliative care in a comparable way. If necessary, we provided some national illustrations mainly through quotations. Furthermore, this paper studies the phenomenon of integration solely from the perspective of professional caregivers who are already involved in palliative care or closely related to palliative care, leaving an opportunity for further research on integration from the perspective of the current medical culture.

## Acknowledgements

 We would like to thank all members of the InSup-C (FP7) consortium for their contributions to this article’s research. In particular, the authors would like to thank Jeroen Hasselaar, Sean Hughes, Agnes Csikos, Karen van Beek, and Lisa Linge-Dahl for their valuable contributions to the quality of the group interviews.

## Ethical issues

 Review committee approvals were obtained in all participating countries. In the Netherlands, the Medical Research Ethics Committee region Arnhem-Nijmegen (https://english.ccmo.nl/mrecs/accredited-mrecs/cmo-region-arnhem-nijmegen) decided the study did not fall within the remit of the Medical Research Involving Human Subjects Act and was therefore waived for further screening by the ethics committee.

## Competing interests

 JvG reports grants from EU Framework 7 Programme, during the conduct of the study (grant agreement: 305555).

## Authors’ contributions

 JvG conceived and designed the analysis; performed the analysis; and wrote the article. JvW conceived and designed the analysis; performed the analysis; and co-authored the article. SP supervised the data collection UK; peer reviewer for analysis and, later, the article. LR supervised the data collection GER; peer reviewer for analysis and, later, the article. KvB collected the data for BEL; peer reviewer for analysis and, later, the article. AC supervised the data collection HUN; peer reviewer for analysis and, later, the article. MHvdE collected the data for NL; performed the analysis; peer reviewer for this article. JH performed the analysis; co-author of the article.

## Authors’ affiliations

^1 ^Department of IQ healthcare, Radboud University Nijmegen Medical Centre, Nijmegen, The Netherlands. ^2^Erasmus School of Health Policy & Management, Health Service Management and Organisation, Erasmus University Rotterdam, Rotterdam, The Netherlands. ^3^International Observatory on End of Life Care, Division of Health Research, Lancaster University, Lancaster, UK. ^4^Department of Palliative Medicine, University Hospital Bonn, Bonn, Germany. ^5^Department of Radiation-Oncology and Palliative Medicine, University Hospital Gasthuisberg, Leuven, Belgium. ^6^Faculty of Medicine, Institute of Family Medicine, University of Pécs Medical School, Pécs, Hungary. ^7^Department of Anesthesiology, Pain and Palliative Medicine, Radboud University Nijmegen Medical Centre, Nijmegen, The Netherlands.

## Supplementary files


Supplementary file 1. Overview of the Group Interview Participants.

Supplementary file 2. Group Interview Protocol.
