# Supplementary material for: Integrating Palliative Care by Virtue of Diplomacy; A Cross-sectional Group Interview Study of the Roles and Attitudes of Palliative Care Professionals to Further Integrate Palliative Care in Europe
Source: Int J Health Policy Manag. 2020 Nov 23;11(6):786–94. doi: 10.34172/ijhpm.2020.211 (PMC9309904; doi:10.34172/ijhpm.2020.211)
Supplement: Supplementary file 1 — Overview of the Group Interview Participants. [file ijhpm-11-786-s001.pdf]

Supplementary file 1. Overview of the Group Interview Participants

Country/number of initiatives	Participants (n)	Functions
Belgium		
- IPC B1	14	Medical oncologist (2); clinical nurse specialist in palliative care (4); district nurse (3); GP; social worker (2); Family care worker (2)
- IPC B2/3	8	Palliative medicine consultant; clinical nurse specialist in palliative care (2); district nurse; GP; social worker; Family care worker (2)
Netherlands		
- IPC NL2	8	Team manager; nursing home physician; physiotherapist (3); pastoral worker; lung care nurse (2)
- IPC NL3	6	GP (2); oncology nurse; care coordinator oncology (hospital); social worker; transfer nurse
- IPC NL4	5	Pharmacist; specialist geriatric medicine; GP; practice assistant for GPs; clinical nurse specialist in palliative care
- IPC NL5	8	Coordinator palliative care network; physiotherapist; district nurse (2); anesthesiologists (2); clinical nurse specialist in palliative care (2)
Germany		
- IPC G1	9	Pastoral worker; physician (2); senior physician; nurses (4); coordinator hospice care service
- IPC G2	6	Nurse (4); physician; GP
- IPC G3	10	Pharmacy employee; psychologist; team administrator; nursing team leader; nurse; care service manager (2); palliative care consultants (2); case manager
- IPC G4	7	Senior physician (2); case manager (2); social worker; coordinator volunteers; physician
United Kingdom		
- IPC UK1	6	Hospice medical director; community matron; clinical nurse specialist in palliative care; GP; hospice spiritual care coordinator; nurse
- IPC UK2	5	Family support team coordinator; home nursing coordinator; district nurses (3); research nurse; GP
- IPC UK3	6	Community matron; clinical nurse specialist in palliative care; district nurse (2); physiotherapist
- IPC UK4	6	GP; hospice staff nurse; hospice; clinical nurse specialist in palliative care; district nurse; palliative care consultant
Hungary		
- IPC D	6	Oncologist; pulmonologist; psychologist (2); head nurse (2)
- IPC B	5	Medical director oncologist; pulmonologist; psychologist; hospice head nurse; palliative care nurse
- IPC P	8	Palliative care physician (2); GP (2); cardiologist; pulmonologist (2); oncology nurse
- IPC M	7	Palliative care nurse; palliative care physician (2); oncologist; pulmonologist; medical director; GP
- IPC K	6	Palliative care nurse; cardiologist; GP (2); specialist internal medicine; palliative care physician
