# Supplementary material for: Integrating Palliative Care by Virtue of Diplomacy; A Cross-sectional Group Interview Study of the Roles and Attitudes of Palliative Care Professionals to Further Integrate Palliative Care in Europe
Source: Int J Health Policy Manag. 2020 Nov 23;11(6):786–94. doi: 10.34172/ijhpm.2020.211 (PMC9309904; doi:10.34172/ijhpm.2020.211)
Supplement: Supplementary file 2 — Group Interview Protocol. [file ijhpm-11-786-s002.pdf]

Supplementary file 2. A Group Interview With Professionals – InSup-C

Who are we going to invite?

- For discussion: various healthcare professionals working in/associated to one particular initiative (inclusion at least partly motivated by patient interviews and Social Network Analyses)

Physical requirements for conducting the interview

- You will need a space that facilitates undisturbed and calm conversations
- The room has to be big enough for +/- 8 people. You arrange the room so as to stimulate interaction (circle; rectangle)
- The room should have a pleasant climate for 90 minutes
- Think about conducting the interview with an independent conversation leader (if available) and one researcher. At least, make sure that the researcher has time/opportunities to bring in his/her particular knowledge about the initiatives.
- What to bring? White board/flip board; markers; audio recorder suited to record group conversations, need a separate, multi directional microphone

---

This interview guide assumes a 90 minute group session

Preparation

- Consider using PowerPoint – or some other visual method – to display the main questions in each of the steps as prompts for the discussion
- Ask participants to complete the demographic sheet in as much detail as they wish

To start: the formal part

- To give everybody a warm welcome
- Explain the aim of the study
- Explain the aim of the group interview:
  "in this interview we would like to explore how you experience, or have experienced working with this/being part of this integrated palliative care initiative .... All of you have

An EU Framework 7 Programme (FP7/2007-2013) under grant agreement n° 335555

somehow been involved in patient care through this initiative, and we would like you to discuss how the multidisciplinary collaboration contributed or, perhaps, impeded high-quality care for the patient. In more general terms: "How is the initiative doing so far?"

- We are especially interested in what goes well in delivering integrated palliative care in the initiative. What are the things they are proud of? Why does that succeed; what can others learn from that?

- We will pose initial questions and/or present propositions to start up the discussion. Please feel free to share your opinions and feelings or to respond to one of the other members of this group. The conversations will be audio-recorded and transcribed. Both the audio files and the transcripts will be kept confidential. Only the researchers have access to the research material. All quotes used in research papers or presentations will be anonymised.

<u>Ground rules</u>

It is useful to have these ground rules on a slide or on flip chart paper for you (and they) to refer to

- Ask participants to switch off phones (or switch to silent if they need to remain available)
- Suggest that if participants need to leave the room (or leave the session) that they do so quietly
- Ask participants to respect confidentiality i.e. not to repeat or talk about what is said in the group interview to others (this is important as many of the people present will continue to work together and it 'frees up' people to be more honest in the discussion)
- Ask participants to treat each other's comments and opinions with respect. It is possible to disagree – but to do so respectfully
- Remind people that the conversation will be recorded and transcribed, so it is important not to speak over each other or at the same time. Tell them that you may remind them of this again if the discussion gets enthusiastic!
- **Before starting the interview, please make an introduction round.**

An EU Framework 7 Programme (FP7/2007-2013) under grant agreement n° 335555

The logic behind the interview

To prepare for the group interview, we suggest that you and the independent chair/conversation leader (re-)read the presentation of Jeroen van Wijngaarden, January 20, 2015, and the article 'Understanding integrated care: a comprehensive conceptual framework based on the integrative functions of primary care' by Valentijn et al. to (re)gain a feel for the dimensions of integrated care. You will need this knowledge of integrated care to check, during the interview, whether participants' answers cover all important dimensions of integrated care. In other words, this knowledge will help you to ask critical probing questions in case of one-dimensional answers.

Also look at the interview material with the patients. Try to identify what they particularly appreciate in this initiative. Present these examples during the group interview to try to understand what is required to get these results.

The interview process

We will invite the group to 'think out loud' about the questions and statements. If a second researcher is present (or a researcher next to the conversation leader), he/she can write key words/phrases on the whiteboard/flipchart so that these may be referred to during the interview: to note links and connections and/or to explore aspects that may have received scant attention.

(While participants are discussing the answers, please stay alert to pick up cues with which you can further the discussion)

Step 1: Description of the integrated palliative care initiative – the patients

a. Please describe a situation in which you believe a patient received good quality integrated palliative care. Why did that go so well?
b. What are in general the things that you are proud of in your initiative? Why does that work so well and what can others learn from that?
c. Please describe a situation in which you believe a patient received poor quality integrated palliative care. Why did that happen and what can we learn from that?
d. What makes the difference between good and bad integrated palliative care?
e. What are the particular 'types' or characteristics of patients for whom integrated palliative care works best/worst?
f. What difference – if any – does the particular condition (cancer, COPD, CHF) make?

An EU Framework 7 Programme (FP7/2007-2013) under grant agreement n° 335555

g. Ideally, at what moment is a patient referred to/admitted into the integrated palliative care initiative?
h. Which professionals are involved with patients at what time(s)?

Try to stimulate discussion as much as possible: ask clarifying questions; ask participants to respond to one another; ask if participants experiences are similar or different. Mentioned below are a few propositions that ***could*** *help stimulate the discussion as well (these propositions contain elements that are available for probing. E.g., the 'how-question'):*

1. Please respond: "Well integrated palliative care covers appropriate assessment at first appointment and on-going review during the disease trajectory."
2. Please respond: "Well integrated palliative care guarantees timely access to services based on patient/family needs and wishes."
3. Please respond: "Well integrated palliative care supports choice and personalized care using tools (where appropriate) such as: advance care plans; advance decision to refuse treatment; do not attempt cardio-pulmonary resuscitation (DNAR) orders; preferred priorities of care etc."

Step 2: Description of the integrated palliative care initiative – communication and information

In this second step, we focus on communication and the sharing of information. Let participants react to the following questions:

1. How does the initiative provide the right information, at the right time?
   a. For patients?
   b. For family caregivers?
   c. For professionals?
   d. What is "the right information, at the right time" (at least)?
   e. How do information needs differ – if at all – between the conditions this study is concerned with (cancer, COPD, CHF)?
2. What communication channels are used by professionals?
3. What are some of the enablers and barriers to good communication?
   a. Between professionals and patients/carers?
      i. How are patients included in care planning and decision making?
      ii. How are family caregivers included in care planning and decision making?

An EU Framework 7 Programme (FP7/2007-2013) under grant agreement n° 335555

b. Between professionals?
   i. How are changes to individual care plans made and communicated to all members of the wider care team
c. Between agencies in the initiative?
d. With other agencies not connected to the initiative **(mentioned in the patient/carer interviews)**?

Mentioned below are a few propositions that ***could*** *help stimulate the discussion as well (these propositions contain elements that are available for probing. E.g., the 'how-question'):*

1. Please respond: "Well integrated palliative care facilitates regular and open conversations about end of life needs, patient values, and patient preferences as well as advanced care planning." + Whether and how should this knowledge be shared with other caregivers.
2. Please respond: "Well integrated palliative care produces information/facilitates appointments about treatment and care during the disease trajectory and towards the end of life – including where the person wishes to die and what services are available at that time and for bereaved people".

You could offer a BREAK to participants if appropriate.

Step 3: Description of the integrated palliative care initiative – the content of care

In this third step, we will focus on the content and continuity of care. Let participants react to the following questions.

a. What makes or who ensures that the patient receives the right level of palliative care at the right moment in the disease trajectory?
b. How is the continuity of care attended to and delivered within the initiative/across care givers and organisations?
c. How is palliative care delivery aligned with the patients' and family caregivers' wishes?
d. How is 'holistic patient assessment' assured? I.e. that all aspects of care are covered: physical, psychological, spiritual and social?
e. What are the local provisions for care overnight and at weekends?

An EU Framework 7 Programme (FP7/2007-2013) under grant agreement n° 335555

Below, you'll find a few propositions that ***could*** *help stimulate the discussion as well (these propositions contain elements that are available for probing. E.g., the 'how-question'):*

1. Please respond: "Well integrated palliative care facilitates open communication about death and dying based on patients and family needs."
2. Please respond: "Well integrated palliative care contains evaluation of pain and other physical and psychological symptoms with adequate access to medications and equipment."
3. Please respond: "Well integrated palliative care contains multidisciplinary care services at generalist and specialist level to provide high quality care at any time during day or night, based on the patient's condition, care plan and wishes."
4. Please respond: "Well integrated palliative care attends to the support needs of bereaved carers".

Step 4: Description of the integrated palliative care initiative – availability of materials and personnel

1. How does 'the initiative' ensure that people with expert knowledge are available to supply the right medication/equipment at the right time?
2. How is the provision of drugs – particularly pain relief and drugs for use at end of life – organised and who takes responsibility for this? **(issues about medication that are raised in the patient/carer interviews)**
3. How is the provision of ADL aids and other equipment organised and who takes responsibility for this?

In addition to the questions/propositions: you could prepare yourself by reading through the interview material and use some striking elements concerning [e.g., patient logistics] to challenge/compliment participants and to further stimulate discussion. If the interview is chaired by another person than the researcher, you'll have to provide the chair beforehand with these 'striking elements'.

<u>**Closure**</u>

An EU Framework 7 Programme (FP7/2007-2013) under grant agreement n° 335555

* Thank the participants for partaking in this focus group. Explain them what will happen from here on with the research material and when and how they will be able to read anything about the research project. If suitable in your country, hand over to the participants the small present for participation and inform them about reimbursement of travel expenses.

Refreshments

Suggestion: read the information below for additional information on what to expect. Some elements could help you to build probing questions or pick critical elements from the interviews. This information also gives you a first insight into the elements we will be looking for in the analysis.

- **Delivery system integration:** the extent at which at the macro level of the care system financing and regulation are aligned for palliative care within and between cure, care and social services. Hindering and stimulating factors at this level can be identified for example:
    - Information logistic may be problematic if regulations do not allow to share patient information across hospital and social care.
    - Integration may be stimulated if cure, care and social services are allowed to transfer and share resources.

- **Functional integration:** The extent at which at the meso level support activities (finance, management and information systems) and organizational structures are aligned for palliative care within and between cure, care and social services.
  What are the structures they introduced at this level or what are the hindering factors; for example:
    - sharing administration to avoid duplication of activities.
    - Introducing an electronic medical record across settings
    - Building an organisation that works across cure, care and social care.

- **Clinical integration:** The extent at which at the micro level care delivery activities are aligned between care givers for palliative care within and between cure, care and social services.
  What has helped integration at this level and what are hindrances;
    - sharing patient information; formal (paper; electronic patient files?) & informal (telephone/teleconsultation/e-mail)
    - multidisciplinary team meetings for decision making (*let participants explain the choices that have been made; let them explain the (potential) value of multidisciplinary team meetings*)
    - patient/family caregiver-inclusive team meetings for decision making (ibid.)
    - collaborative interventions at a patient's bedside (ibid.)
    - *protocols/pathways*

An EU Framework 7 Programme (FP7/2007-2013) under grant agreement n° 335555

- (therapeutic) continuity for the patient
- attuned care for the patient
- collaboration with the patient/family caregiver

- **Cultural integration**: The extent at which norms, values and approaches of care givers are aligned.
  What helped them align norms and values or where do they experience problems.
  - training activities and evaluations; within and between disciplines/institutions (ibid.)
  - a shared philosophy on paper; a signed mission statement. (What did your initiative do to co-create such a philosophy?)
  - collaboration towards a shared philosophy in practice. (What has been done to implement the philosophy into daily practice? How did that work out?)

- **Social integration:** The extent at which social relations between caregivers are intensified; trust.
  What did they do to intensify relationships and trust, where do they experience problems?
  - visits; internships, meetings.
  - concrete definitions of the different responsibilities and roles of the various professionals/volunteers/patients/family caregivers
  - versatile and flexible professionals who can build on each other

- **Strategic integration:** The extent at which goals, means, power and interests of organizations and caregivers are aligned.
  What did they do to align goals, means, power and interests. Where do they experience problems?
  - Hired an independent project coordinator
  - Used a specific implementation strategy.

An EU Framework 7 Programme (FP7/2007-2013) under grant agreement n° 335555
